# Neighborhood Socioeconomic Status and Women’s Mental Health: A Longitudinal Study of Hurricane Katrina Survivors, 2005–2015

**DOI:** 10.3390/ijerph20020925

**Published:** 2023-01-04

**Authors:** Angela-Maithy Nguyen, Yeerae Kim, David M. Abramson

**Affiliations:** 1 Berkeley School of Public Health, University of California, Berkeley, CA 94704, USA; 2 School of Global Public Health, New York University, New York, NY 10003, USA

**Keywords:** disaster mental health, natural disasters, disaster recovery, disaster mental health, Hurricane Katrina, neighborhood effects

## Abstract

There is limited knowledge on the relationship between neighborhood factors and mental health among displaced disaster survivors, particularly among women. Hurricane Katrina (Katrina) was the largest internal displacement in the United States (U.S.), which presented itself as a natural experiment. We examined the association between neighborhood socioeconomic status (SES) and mental health among women up to 10 years following Katrina (N = 394). We also investigated whether this association was modified by move status, comparing women who were permanently displaced to those who had returned to their pre-Katrina residence. We used hierarchical linear models to measure this association, using data from the American Community Survey and the Gulf Coast Child and Family Health study. Neighborhood SES was created as an index which represented social and economic characteristics of participants’ neighborhoods. Mental health was measured using mental component summary (MCS) scores. Increased neighborhood SES was positively associated with mental health after controlling for age, race/ethnicity, economic positioning, time, and move status (19.6, 95% Confidence Interval: 5.8, 33.7). Neighborhood SES and mental health was also modified by move status. These findings underscore the need to better understand the impacts of socioeconomic conditions and health outcomes among women affected by natural disasters.

## 1. Introduction

Existing research has established links between place and health, in that the neighborhood context plays a pivotal role in influencing health outcomes. Among the conceptual underpinnings of this association between place and well-being are (a) social determinants of health, (b) fundamental cause (access to power, resources, status), (c) economic and social opportunity, (d) social and cultural norms, (e) environmental hazards and toxins, and (f) structural and instrumental assets or liabilities, among others, in addition to a broad range of individual-level characteristics [1,2,3]. While the majority of previous studies have focused on physical health outcomes, there is some evidence to suggest that the neighborhood factors may impact mental health outcomes both in the short- and long-term, including outcomes related to overall self-rated mental health, depression, psychological distress, and anxiety [1,4]. The broader literature on neighborhood and health outcomes is indeed vast; however, the impacts of neighborhood environment among vulnerable and displaced populations remain underexplored. This study seeks to bridge this gap by examining the association between neighborhood socioeconomic status and mental health over time among women survivors of a natural disaster.

Some of the earliest research on neighborhood factors and mental health among women came from the Stirling County Study, a prospective epidemiological study conducted in Atlantic Canada in the 1950s following the Second World War [5,6]. The incidence of depression among women were double that of men. These findings generated the hypothesis that changes in the post-war social environment related to educational opportunities, family ties, racial tensions, employment, and poverty led to disintegration and were associated with poor mental health. Furthermore, these findings garnered interest to better understand how environmental conditions, in addition to individual-level socioeconomic positioning and life experiences, were important for women’s mental health. A more recent study found that the residential environment was likely to be more important for women’s self-rated health than it was for men, specifically characteristics related to the physical environment and access to economic opportunities [7]. In addition, a qualitative study was conducted among African American women who participated in the Moving to Opportunity experimental study to better understand the processes of how moving to a low-poverty neighborhood impacted their mental health [8]. The study’s findings were that social environmental factors related to collective efficacy, physical environmental factors related to improved neighborhood, and home aesthetics were important for mental health. While gender differences may arguably be context or population specific, these limited research findings suggest that the neighborhood environment has significant impacts on women. However, most studies have either treated gender as a confounder in analytic models and/or have been cross-sectional in nature, warranting further investigation. 

There is also limited literature on the relationship between neighborhood effects and health outcomes among women who have experienced natural disasters. The relationship between place and health is crucial in the context of post-disaster mental health for several reasons. First, in the event of a natural disaster, forced migration or displacement is likely to occur. In the case of Hurricane Katrina, the disaster resulted in the displacement of over 400,000 residents in New Orleans, either temporarily or permanently. Hurricane Katrina also directly affected structurally vulnerable regions of the United States. In New Orleans, approximately one-third of the population was living below the poverty line at the time of Hurricane Katrina [9]. Due to the significant displacement, residents were forced to resettle in new communities. Second, neighborhoods themselves experience significant changes post-disaster due to disruption, rebuilding, and recovery processes [10,11]. Geographic mobility, neighborhood changes, and social ties are all important factors related to disaster recovery.

This research study is guided by the Fundamental Cause Theory and the Spatial Opportunity Structure Framework [12,13]. Link and Phelan developed Fundamental Cause Theory to better understand how to contextualize risk factors as a process and to determine the social conditions under which people are “at risk of risk”. Fundamental causes, such as socioeconomic status (both at the individual or community level), are associated with access to flexible resources which allow people to minimize their risk or maximize their protection. These flexible resources include power, prestige, money, as well as interpersonal resources, such as social capital, and these flexible resources are embedded in neighborhoods. People’s access to flexible resources are bound to change when their neighborhood environments are changing. 

The mechanisms in how neighborhood social and economic conditions influence health may also be best understood through a proposed “Spatial Opportunity Structure” framework [13]. The spatial opportunity structure is thought of as variations in natural or intentional spatial inequalities in socioeconomic, environmental, institutional, and political domains. This conceptual framework is modeled as several pathways between an individual’s life decisions, individual’s attributes, spatial opportunity structure, and individual’s achieved status. An individual’s attributes include both fixed and malleable characteristics over a lifetime. First, spatial opportunity structures are thought to act as a mediator between individual’s current characteristics and desired outcomes. One’s access to opportunities and ability to “convert their most productive personality attributes” will always be influenced by where they live, work, and socialize. Second, spatial opportunity structures may also function as modifiers through environmental exposure. Physical and/or social environmental factors may unconsciously shape or change individual-level attributes, behaviors, or preferences. Some examples include exposure to neighborhood violence or collective socialization. Spatial opportunity structure influences how individuals make life decisions through shaping how individuals perceive their ability to access and/or receive information. When it comes to decision-making, individuals are influenced by what they perceive are the more desirable and feasible options for them. These pathways and processes are viewed cumulative across the life course and mutually reinforcing over time. Given the complexity of this model, estimating the causal impacts of spatial context is challenging due to unmeasured confounding, geographic selection bias, timing of events, and instrumental variables for spatial context characteristics. Applying both the Fundamental Cause Theory and the Spatial Opportunity Structure Framework, this study aims to examine the impact of neighborhood socioeconomic status on mental health among women survivors of Hurricane Katrina over time through a multilevel framework.

The overarching purpose of this study is to understand how changes to the social and economic environment shape mental health trajectories among women survivors of Hurricane Katrina, using data from the Gulf Coast Child and Family Health Study (GCAFH). In this longitudinal study, we examined the association between neighborhood socioeconomic status and self-rated mental health among Hurricane Katrina women survivors, and whether these associations were different between those who were permanently displaced and those who stayed or returned to their pre-disaster residence. To our knowledge, there are no longitudinal studies that have investigated neighborhood effects on women’s mental health trajectories. Given similar pre-Hurricane Katrina neighborhoods and household economic positions, we hypothesized that increasing neighborhood socioeconomic status would be associated with improved mental health in our study population of women survivors of Hurricane Katrina. 

## 2. Materials and Methods

### 2.1. Individual-Level Data and Study Sample

This study used data from the GCAFH study, a longitudinal cohort of Louisiana and Mississippi households impacted by Hurricane Katrina (Katrina). The cohort comprises of 1079 households randomly sampled from the Federal Emergency Management Agency’s list of group sites, commercial trailer sites, and hotels. Cluster stratified sampling using a probability proportional to size strategy without replacement was used to produce a representative GCAFH cohort of the approximately 50,000 individuals who had been displaced in Louisiana and Mississippi, in addition to approximately 26,000 people who were living in the severely damaged areas of the Mississippi Gulf Coast after Hurricane Katrina. A baseline survey was conducted 6–12 months post-Katrina (Wave 1) and four follow-up surveys were conducted in 2007 (Wave 2), 2008 (Wave 3), 2009 (Wave 4), and 2015 (Wave 5). The survey includes a range of measures related to physical and mental health, recovery, mobility, social ties, and individual-level demographics and socioeconomic status. Detailed descriptions of the study and recruitment process of the GCAFH cohort have been previously provided [14].

Our inclusion criteria for our study sample were participants who completed Wave 1, Wave 5, and at least one other wave (Waves 2, 3, or 4). In order to conduct a longitudinal analysis using a hierarchical model, our analysis required at least three data points [15]. We then restricted our sample to women, then further restricted our sample to include participants with complete residential addresses provided during each survey completion for geocoding purposes. We also limited the study sample to those with available data on the outcome of interest, mental component summary (MCS) score. This resulted in a final analytical sample of 394 participants. 

#### Outcome Measure

Our outcome of interest was self-rated mental health as derived from a 12-item Short Form Health Survey (SF-12) composed of several physical and mental health domains including physical functioning, role-physical, general health vitality, social functioning, role-emotional, and general health. The SF-12 is a subset of the 36-item Short Form Health Survey (SF-36) and is a validated instrument to measure health-related quality of life measures [16]. The instrument generates two summary scores known as the Physical and Mental Component Summary Scores (PCS and MCS, respectively) [17]. Of the 12 sub-items on the SF-12, six of them make up the MCS. These six sub-items are related to how much time, in the last four weeks, an individual felt they were able to accomplish tasks, perform work, socialize, and their perceived levels of mental health symptoms and vitality. Scores range from 0 to 100, where higher scores represent overall better mental health. The SF-12 was administered at each survey wave, and we used continuous MCS scores as our outcome measure for self-rated mental health.

### 2.2. Population-Based Data Sources

To examine the social and economic characteristics of neighborhoods, we used census block group level information from the American Community Survey (ACS), an ongoing survey which covers topics about social, economic, demographic, and housing characteristics of the U.S. population [18]. We selected five-year estimates closely aligned with GCAFH Waves 1–5 surveys (2005–2009, 2006–2010, and 2012–2016). Five-year estimates represent 60 months of collected data. We chose to use five-year estimates due to their statistical reliability for examining less populated areas or geographical units, such as census tracts or census block groups. We also obtained data from the Longitudinal Employer-Household Dynamics (LEHD) Origin-Destination Employment Statistics (LODES) [19]. We extracted LODES data on the total number of jobs within five miles of census block groups of interest for years 2005, 2007, 2008, 2009, and 2015.

#### Exposure Measure

We defined neighborhoods using census block groups, statistical divisions of census tracts which contain between 600 and 3000 people. We geocoded GCAFH participants’ addresses which were reported during each survey completion. Geocoded addresses were then matched to 2000 and 2010 census block group boundaries using spatial files provided by Integrated Public Use Microdata Series (IPUMS) National Historical Geographic Information Systems (NHGIS) [20]. The spatially joined data were then linked to ACS data. The baseline GCAFH survey data, Wave 1, was linked to ACS 2005–2009, Waves 2–4 were linked to ACS 2006–2010, and Wave 5 was linked to ACS 2012–2016.

The exposure measure, neighborhood socioeconomic status (SES), was conceptualized to represent a range of social and economic factors identified a priori, specifically referencing the neighborhood characteristics from the broader literature on measuring neighborhood-level SES [21,22,23]. We selected 10 sub-items to create our measure of neighborhood SES. These sub-items were social and economic factors related to racial and ethnic composition (percentage of non-Hispanic residents and percentage of White residents), head of household (percentage of dual-parent households), housing tenure (percentage of home owners), immigrant status (percentage born in the United States), educational attainment (percentage with Bachelor’s degree or higher), percentage above the federal poverty line, employment status (percentage employed), median household income (MHI), and short work commute time (percentage of residents who commute fewer than 15 min to work). We also included population density, calculated as the total number of people in the census block group divided by the land area (square miles). All calculations were conducted for each United States census block group for each of the five Waves and then merged with the geocoded GCAFH residential address data. We had considered two additional variables, residential stability (percentage of residents who lived at the same residence address within the past year),, and job density (total number of jobs in a census block group divided by land area), but they were subsequently removed because the variables at the time had a large proportion of missing data at the census block group level. We created a summary score measure for neighborhood SES via confirmatory factor analysis (CFA) with the 10 identified sub-item census block group-level variables related to neighborhood social and economic factors. CFA was used to test our hypothesis on whether an underlying factor explained the correlations among the selected observed variables. A summary score was generated for each individual at each completed Wave to summarize their neighborhood-level SES at a given time point. The factor scores were generated to have a mean of 0 and standard deviation of 1, which were then standardized to range from 0 to 1 and was scaled such that a higher score represented increasing SES within the neighborhood. Confirmatory factor analyses were conducted using the R package ‘lavaan’ [24].

### 2.3. Additional Covariates

Several individual-level characteristics were considered as potential confounders between neighborhood SES and mental health. We included age at the year of Katrina, race/ethnicity, presence of children in the household, and physical component summary (PCS) score as sociodemographic- and health-related covariates in our analyses. We hypothesized that children under 18 in the household was an important factor considering women are key decision makers and may have different motivations about where they relocate across different life stages [25]. In addition, the literature indicates that parents are vulnerable to poor mental health outcomes after natural disasters [26,27].PCS scores were obtained via the SF-12 scale which examines constructs related to physical functioning, role-physical, and general health. Similar to MCS scores, PCS scores range from 0 to 100, 100 representing perfect health. We also included number of months since Katrina as a time variable, household relative poverty status, and household relative income status. We defined household poverty as a participant’s household income relative to the federal poverty threshold during each survey year. We created a dichotomous variable to indicate whether a participant’s self-reported household income was below (1 = yes) or not below (0 = no) the federal poverty threshold. Household relative income was also defined as a dichotomous variable to indicate whether a participant’s self-reported household income was below (1 = yes) or not below (0 = no) their neighborhood-level MHI. We also included variations of several variables to capture a participant’s status at baseline/Wave 1 and before Hurricane Katrina (pre-Katrina). These variables included pre-Katrina neighborhood-level SES, baseline household relative poverty status, and baseline household relative income status. Lastly, we created a categorical variables related to participants’ move status. We created a dichotomous variable, move status, to indicate those who stayed or returned to the original area of residence at any given timepoint following Katrina (=0) and those who moved and never returned to the original area of residence following Katrina (=1). The variable was created by comparing the participants’ self-reported addresses at pre-Katrina and at Wave 5. We adjusted for all the covariates in our intermediate and final analytical models.

### 2.4. Statistical Analyses

A three-level multilevel modeling approach was used to examine the relationship between neighborhood SES and self-rated mental health. Repeated measures of mental composite health scores over time (*i*) were fully nested within individuals (*j*), while the neighborhoods determined by census block groups (*k*) were treated as crossed with individuals since the individuals could have moved to different neighborhoods between Waves (Equation (1)). Our general model is as follows:

(1) $$y_{ijk}=\beta_{1}+\beta_{2}x_{j}+\zeta_{j}^{\left(2\right)}+\zeta_{k}^{\left(3\right)}+\varepsilon_{ijk}$$

$\zeta_{j}^{\left(2\right)}\sim \;N\left(0,\psi^{\left(2\right)}\right)$: Random intercept for individual *j*;

$\zeta_{k}^{\left(3\right)}\sim N\left(0,\psi^{\left(3\right)}\right)$: Random intercept for neighborhood *k*;

$\varepsilon_{ijk}\;\sim \;N\left(0,\theta \right)$: Error term at occasions *i* for individual *i* and neighborhood *k*.

We used random intercept models to test the main study hypothesis. Our initial exploratory analyses, model building and final model selection came in several stages. First, visual exploratory analyses were conducted by generating empirical growth plots as well as average change trajectories of the sample to assess how each subject changed over time. Age, as well as the time variable measured in months between Katrina and survey dates, were centered to facilitate the interpretation of results. Second, we fitted an unconditional means model with no predictors to estimate the variance components. Intraclass correlation coefficients (ICC) were calculated to describe the proportions of total variation that were explained by within-subject, between subject, and between block group variation. Next, we ran an unconditional growth model, including the time variable measured by number of months since Katrina. Then, to understand if there was a difference in the rates of change in MCS scores based on the changes in the neighborhood SES scores, an interaction term between the neighborhood SES scores and the time variable was fitted into the model. However, the interaction was not statistically significant. Thus, we built the intermediate model in which we controlled for age, race/ethnicity, PCS score, move status, household relative poverty status, household relative income status, any children under 18 years old present in the household at baseline, and time. We also included contextual factors at pre-Katrina: neighborhood SES score, household relative poverty status, and household relative income status. In addition to these factors being potentially important factors to control for, we were interested in estimating their independent associations on mental health over time. Lastly, we included an interaction term in our final model to assess whether the variable move status modified the association between neighborhood SES and mental component score. Our final model also adjusted for all the same covariates as the intermediate model.

Restricted maximum likelihood estimation was used for all models, and comparative model fit statistics were calculated using likelihood ratio tests for nested models and Akaike Information Criterion for non-nested models (AIC). Statistical significance was determined at an alpha level of 0.05. All descriptive and statistical analyses were conducted using ArcMap 10.6.1, Stata IC 16.1, and R v. 3.6.1. [28,29,30].

## 3. Results

### 3.1. Confirmatory Factor Analysis

We used a one-factor model using the percentages of non-Hispanic White, two-parent households, homeownership, US-born, college education, above poverty line, employment, MHI, short work commute time and population density as our neighborhood-level variables manifest variables to indirectly measure the latent variable, neighborhood SES. We inspected estimated factor loadings from our model to assess the association between the manifest variables and the latent variable. Among the 10 manifest variables used to create neighborhood SES, we found that living above the federal poverty line, two-parent household, homeownership, White, MHI, education, employment, short travel time and US-born were positively associated with the latent variable (standardized loading factors were between 0.5 and 0.9 for each indicator). The remaining manifest variables, population density, was negatively associated with the latent variable (standardized loading factor −0.85). In order to assess how well our data fit the specified one-factor model, we examined several commonly reported approximate fit indices [31]. Given the increasing controversies surrounding cutoff values for CFA models, we assessed our approximate fit indices with caution [32,33]. Comparative Fit Index (CFI) and the Tucker–Lewis Index (TLI) values close to 1.0 are generally preferred. Our results demonstrated an adequate fit based on our CFI (0.9) and TLI (0.9) results. We also examined the Root Mean Square of Error of Approximation (RMSEA) and Standardized Root Mean Square Residual (SRMR), non-centrality indices, where values closer to 0 support acceptable fit. Our model SRMR and RMSEA values were 0.08 and 0.10, respectively.

### 3.2. Descriptive Findings

The analytical sample included 394 women. Table 1 shows the baseline characteristics of the study samples stratified by the variable move status. Overall, the mean age of women was 42.7 years (Standard Deviation (SD) = 13.8) and their average MCS score was 40 (SD = 10.7). More than half of the women were Black (52%), non-partnered (62.4%), and had at least 1 child in the household at baseline (60.7%). At the pre-Katrina household level, more than half of the women were below the poverty line and over 70% of them had a household income below their pre-Katrina neighborhood MHI. Most women (82.5%) moved out of their pre-Katrina neighborhood and did not return. Those who stayed or returned tended to be older (48.4 years, SD: 13.5) compared to those who moved and did not return to their pre-Katrina neighborhood. Nearly 60 percent of those who permanently moved out of their pre-Katrina neighborhood were living below the poverty line and over 70 percent had a household income below their pre-Katrina’s neighborhood’s MHI. A higher proportion of those who either stayed or returned were partnered (48.4%) compared to those who moved and did not return (35.3%). The mean neighborhood socioeconomic score of pre-Katrina neighborhoods was higher among those who either stayed or returned (0.57, SD: 0.13) compared to those who moved and did not return (0.53, SD: 0.14).

**Figure 1:**
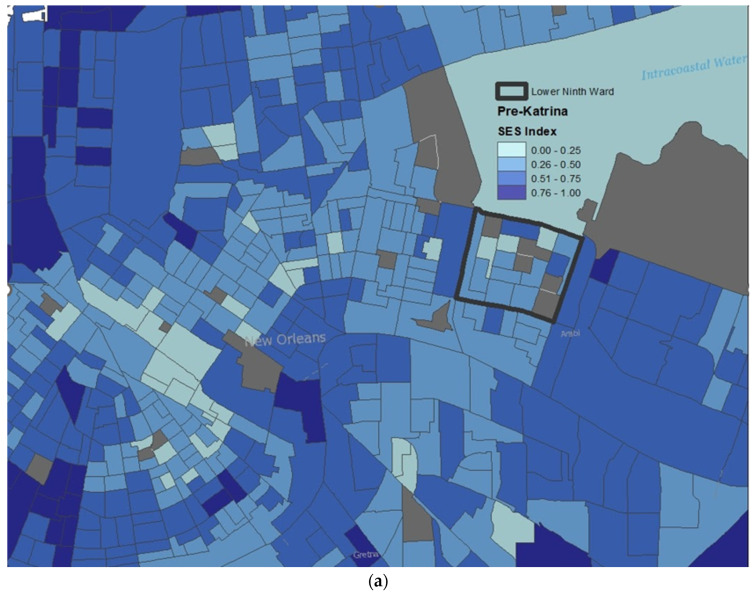

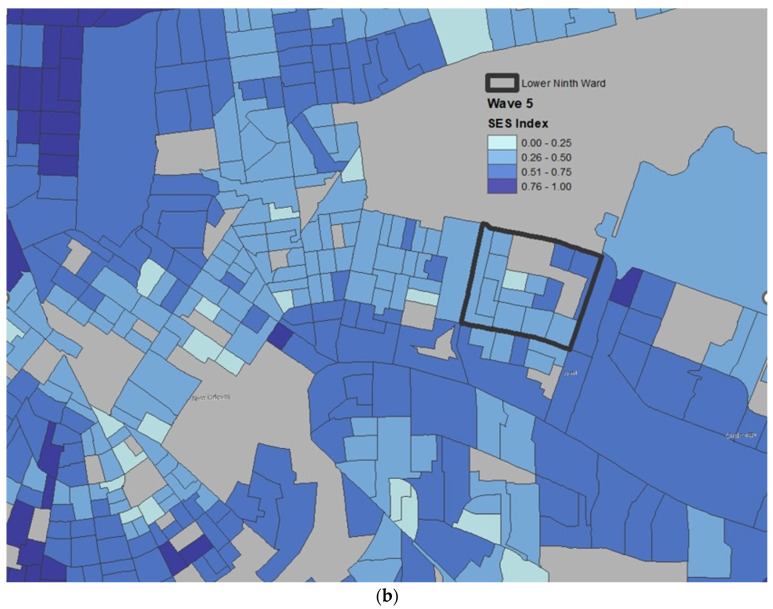
Thematic visualization of neighborhood socioeconomic index (SES) score in New Orleans at (**a**) Pre-Katrina (2005) and at (**b**) Wave 5 (2015). Darker gradients suggest relatively increased SES at the census block group level.

A thematic depiction of the distribution of and changes in neighborhood socioeconomic scores is presented in two choropleth maps shown in Figure 1. These maps visualize the derived neighborhood socioeconomic scores during pre-Katrina (a) and at Wave 5 (b) for a sample of census block groups in downtown New Orleans. Areas shaded in grey are census block groups in which we were not able to produce scores due to incomplete data for one or more indicator variables. Neighborhood socioeconomic index scores were categorized into quartiles, with darker shades representing high socioeconomic scores (relatively increased SES) and lighter shades representing low socioeconomic scores (relatively decreased SES). While some census block groups in New Orleans may have increased in terms of socioeconomic status, this does not necessarily translate to increased socioeconomic status for Katrina survivors many of whom in our study population had moved away from the city.

### 3.3. Main Findings

#### 3.3.1. Unconditional Means and Growth Models

Table 2 presents our results from the initial exploratory models, the unconditional means and growth models. The unconditional means model, with no predictors included, shows that the average mental health scores for women in our sample across all time points and all observations was estimated as 44.5. The total variance is largely driven by within-individual variance followed by between individual variance and between block group variances. The ICCs show that 31% of the variability is attributable to between individual characteristics while about 9% of the variability is attributable to census block group characteristics. The estimated variance components showed that there is sufficient variation at each level to warrant further analysis. The unconditional growth model (Table 2) was fitted by adding the time variable as the number of months since Hurricane Katrina. Compared to the unconditional means model, a better model fit was achieved by adding the time variable into the model. The slopes were statistically significant, with mean MCS scores estimated to increase by 0.04 for every month post-Katrina. The estimated variance components for census block groups decreased significantly. The declined variances were also reflected in the significant decrease in ICC for neighborhood variability. The unconditional growth model demonstrated that time is a significant factor in explaining MCS trajectories for the study population.

#### 3.3.2. Intermediate and Final Models

The results of the intermediate and final models are presented in Table 3. Compared to the unconditional growth model, the within-individual variance estimated in the intermediate model decreased from 97.7 to 90.7. This is expected because the model includes significant time-varying variables including PCS score, living below the poverty line at any wave, and having a household income below the neighborhood MHI. Despite adding time-invariant variables in the model (age, race, and any child in the household at baseline), the between-individual variance estimated did not decrease compared to results from the unconditional growth models. Estimates of the census block group variance as well as between-person variance decreased in the final model compared to the intermediate model. Similarly, the ICC for neighborhood variability also decreased, demonstrating the decreased variability attributable to neighborhood characteristics. Lastly, the AIC was slightly lower for the final model compared to the intermediate model.

On average, MCS scores increased over time in both the intermediate and final models when adjusted for neighborhood SES score at pre-Katrina, neighborhood SES scores at any wave, age at baseline, race, any child under 18 years old in the household, PCS, living below the poverty line at pre-Katrina, living below the poverty line at any wave, having a household income below the neighborhood MHI at pre-Katrina, having a household income below the neighborhood MHI at any given wave, and move status. Neither age nor race was an important predictor of MCS in any of the models. PCS score and having a household income below the neighborhood MHI were statistically significant predictors in both the intermediate and final models. For every one unit increase in PCS score, MCS scores were estimated to decrease by an average of 0.02 among women. As for relative economic positioning, women’s households below neighborhood MHI was statistically negatively associated with MCS score.

**Figure 2:**
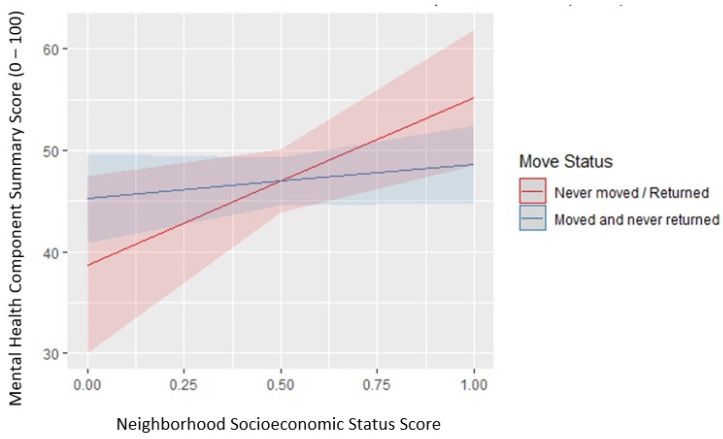
The effects of neighborhood socioeconomic status score on predicted values of mental health scores by move status.

The exposure of interest, neighborhood SES, was positively associated with MCS in our intermediate model though not statistically significant. The confidence interval indicates this association is partially significant. The results from the final model for our analysis, in which we assessed for interaction between residential mobility and neighborhood SES score, are shown in Table 3. In this final model, we observed a statistically significant coefficient for neighborhood SES score, after including the interaction term for move status. For those who did not move or eventually returned to their pre-Katrina neighborhood, every unit increase in neighborhood SES score was associated with an estimated increased mean MCS score by 19.6 (95% CI: 5.8, 33.7). In other words, mental health score improved as neighborhood SES increased for those who did not move or eventually returned to their pre-Katrina neighborhood, controlling for all other variables. The interaction term between neighborhood SES score and move status was statistically significant, suggesting that the effect of neighborhood SES on MCS was modified by move status. The coefficient for this interaction term, −13.14 [95% CI: −27.92, 1.63], further indicates that the positive relationship between neighborhood SES and MCS was attenuated among those who moved and did not return compared to those who did not move or eventually returned to their pre-Katrina residence. Based on the *p*-value of the interaction coefficient, the effect of neighborhood SES on mental health was modified by move status. To further interpret these findings in a more tangible sense, Figure 2 presents a marginal effects plot to visualize this interaction, how the estimated effect of neighborhood SES varies by move status. A marginal effects plot allows one to interpret results from the statistical model by presenting the estimated predicted values generated by the model [34]. These predictions compare how the outcome, MCS score, changes on average, holding the non-focal variables constant. In this figure, MCS scores increase over time for both slopes. Regardless of move status, mental health was predicted to improve for both groups. However, the slope of those who never moved or returned to their pre-Katrina neighborhood is steeper than the slope of those who moved and never came returned, emphasizing the significance of the interaction. Those who never moved or returned to their pre-Katrina residence have a predicted higher average rate of increase in MCS score (mental health improvement) than those who moved away from their pre-Katrina residence in the 10 years following Hurricane Katrina. Our final model demonstrates that the effect of neighborhood socioeconomic status is different for those who moved away than those who never moved or eventually returned to their pre-Katrina neighborhoods.

## 4. Discussion

This study examined whether neighborhood SES was positively associated with self-related mental health among women survivors up to 10 years after Hurricane Katrina. To our knowledge, this is the first study to examine the impacts of SES among disaster survivors, particularly the independent impacts among women, most of whom experienced post-disaster displacement. This research study extends the work of previous research by (1.) examining the association of neighborhood SES and mental health over a 10-year period among disaster women survivors and (2.) investigating how these associations differ by post-disaster move patterns.

There were three main findings from our research study. First, increased neighborhood SES is associated with improved mental health among women, which supported our main hypothesis. Second, our interaction analysis indicated that women who stayed or returned to their pre-disaster area of residence, compared to those who never returned, were more likely to experience better mental health living in higher socioeconomic conditions. Third, women’s household economic positioning was associated with decreased mental health.

Our first main finding is novel and adds to the growing literature on how neighborhood factors shape mental health among women survivors of a natural disaster. Recent systematic reviews on neighborhood effects and mental health indicate that previous studies have generally only controlled for gender, while our study was focused on women [1,4,35]. Findings from an early cross-sectional study in a rural community found that women’s perceptions of the social quality of their community (i.e., access to health care, quality of public education, crime) were positively associated with their self-reported physical and mental health status and daily functioning [36]. In contrast, perceptions of the physical environment (i.e., air quality, drinking water, waste disposal) were significantly associated among men. A later study also found significantly larger between-neighborhood differences in self-rated health among women than for men [7]. More specifically, women’s self-related health was more strongly associated with their perception of their neighborhood’s socio-political, physical, and economic characteristics compared to men. Some plausible explanations for gender differences are that (1.) men and women perceive their environment differently, (2.) exposure to various aspects of the local environment may differ between men and women, and (3.) the vulnerability to the aspects of the local environment may differ between men and women. Gender differences may also intersect across adult life stages. Another explanation is that there are different levels of exposure to the neighborhood between women and men, in that women may spend more time in the neighborhoods than men, but there is no empirical evidence to support this theory. While findings on gender differences in neighborhood effects are limited, research on gender differences in the context of natural disasters is even more scarce. Before Katrina, many Gulf Coast women lived in environments, with poor access to health care, limited healthy food and recreational opportunities, and proximity to crime and violence [37,38,39]. Katrina re-shuffled the deck, displacing many women from their pre-Katrina neighborhoods. The neighborhoods to which they moved varied considerably along the dimensions of social and economic status, as well as such structural harms as inequality, segregation, and deficits of social capital [40]. Though not directly related to the spatial contexts of SES, one study’s findings showed that community-level social support was a strong predictor of long-term happiness among women exposed to Katrina [41]. These findings underscore the need to better understand the impact of neighborhood factors and mental health among women affected by natural disasters, specifically neighborhood-level factors more closely aligned with social capital, crime, and access to health services (both objectively and subjectively).

Our second finding on move status as an effect modifier suggests that women’s displacement patterns are an important factor on their mental health over time, in conjunction with their access to a more socioeconomically advantageous neighborhood. Natural hazards which displace large numbers of people hold the potential to serve as natural experiments to understand neighborhood effects on health among community survivors. Katrina was the largest displacement event in modern United States history. After Katrina, survivors were displaced, at least temporarily from New Orleans, and had little or no control over where they were able to relocate to immediately following the storm. This near-random displacement after Katrina created a natural experiment [42]. A study on displacement patterns among low-income parents (predominantly single mothers) found that participants who returned to their pre-disaster communities had lower levels of psychological distress compared to those who were permanently relocated or in unstable housing [43]. In our intermediate model, we observed that increased neighborhood SES score was positively associated with increased mental health over time, though this association was only partially significant. In our study’s final model, we tested whether the effect of neighborhood SES on mental health over time was different between those who never moved or eventually returned to their pre-Katrina residence and those who moved away and did not return. We found that this difference between types of movers was significant. In short, those who never moved or eventually returned to their pre-Katrina residence were predicted to have a higher average rate of mental health increase compared to those who never returned. Our findings suggest that, while everyone in our study sample may potentially benefit from increased neighborhood SES, those who were able to return to their pre-disaster neighborhoods experience greater improvements in their mental health. It is likely that those who were able to return to their pre-disaster communities reconnected with or had stronger social support networks, which aided their mental health. Individuals who returned to their pre-disaster communities, particularly areas which were relatively more impacted by the hurricane, may have benefited from the reinvestment and rebuilding of neighborhoods. Though not everyone had an equal opportunity to return. Black and low-income residents returned to New Orleans at slower rates than White individuals and the city became “older, richer, and more White than it was before Katrina” [37]. Our descriptive findings indicate that those who eventually returned to their pre-Katrina residences were likely to have more economic resources to do so. Further exploration of the lived experiences of those who were displaced is needed, particularly the decision-making processes behind relocating and returning.

Our third finding is important in understanding the impact of women’s household’s post-disaster economic positioning, relative to where they live or the poverty thresholds, on their mental health. We examined the relationship between two relative SES measures and mental health, (1.) an individual’s household income relative to their neighborhood’s MHI and (2.) whether an individual’s household income was above or below the federal poverty line. Women’s household economic positioning relative to their neighborhood or the federal poverty line prior to Katrina was not significantly associated with their MCS scores. However, women having a household income below the neighborhood MHI at any given time point was a significant predictor for declining mental health score. These findings suggest that economic positioning for women’s households relative to where they are living after a disaster has significant impacts on their mental health both in the immediate and long-term. Additionally, overall, these results further support the theory of relative SES as an important social determinant of health. While the SES gradient of health and the dimensions of individual SES have been well documented, the relationship between community socioeconomic conditions and health is inconsistent [44,45,46]. Our findings warrant further investigation on the role of socioeconomic positioning relative to one’s neighborhood across the life span, particularly among disaster survivors who were forced to relocate and adapt in new places.

Our study was subject to several limitations. We utilized a multi-level framework with the assumption that there was a level of heterogeneity across neighborhoods. While mixed effects models offer flexibility, results should be approached with caution because mixed effects models involve normality assumptions for the error distribution. We were also not able to examine the length of residence at each participant’s address(es) nor the number of times people moved. Long-term residence may be associated with health outcomes as it relates to mechanisms of social integration and social ties [47,48]. Frequent moves and temporary housing may also impact mental health [37,43]. As previous studies have shown, individuals’ subjective experiences or perceptions of the neighborhoods in which they reside play an important role in their health. We utilized an objective measure to conceptualize the latent construct of neighborhood SES, which might differ from how individuals perceive their access or potential access or from the resources that are available to them personally. We were limited in using the 2006–2010 5-year ACS estimates (2006–2010) for constructing the census block group-level index for Waves 2–4, thus we were not able to capture any actual neighborhood changes among participants with the same residential address for those given Waves. This implies that our measure may be underestimating the impact of neighborhood socioeconomic changes on health. Additionally, there is no gold standard in neighborhood SES, thus our exposure may not be precise. There were other factors we were interested in including in our index, but we were met with challenges in obtaining publicly available data for the years and geographic scale of interest. However, given the limitation in census data, we were able to include the majority of variables referenced a priori. We measured our outcome of interest, mental health, using the SF-12 MCS, which is a subjective measure and may result in response bias. Furthermore, this measure does not assess for probable diagnosis of a mental health condition but instead measures specific domains of mental health and well-being. Lastly, our study findings may not be generalizable to the general population. The replication of our study’s methodology both for Katrina and other large-scale catastrophes is critical to (1.) test the robustness of our results and (2.) increase our overall understanding of the effects of neighborhood among disaster survivors.

Nonetheless, our study is one of the first studies to examine the relationship between neighborhood SES and mental health among natural disaster survivors over time. Our study also contributes to the limited knowledge on how these neighborhood effects impact women survivors. Methodologically, our study has several strengths. First, we were able to assess changes over time with multiple Waves of data up to 10 years following Katrina. We used several rigorous methods to operationalize our exposure and model our research questions. By utilizing a multilevel model approach, we were able to account for intra-individual differences and observe neighborhood-level effects on individual-level outcomes. Our exposure variable, neighborhood SES, was a robust measurement scale that included 10 census block-group level variables related to neighborhood social and economic factors. Our method in using confirmatory factor analysis builds upon the existing SES and health literature to develop and refine our latent construct of interest. We also had information on each participant’s pre-Katrina address, which allowed us to examine both the movement trajectories and neighborhood changes our study population experienced before, during, and after the disaster. Few studies have any pre-disaster address data, due to the unpredictability of natural disasters, which makes it challenging to examine the extent of these kinds of changes [49]. We were also able to control for baseline neighborhood conditions and other economic variables including relative neighborhood income and economic deprivation, some of which had statistically significant impacts on mental health.

Our study has several important implications for policy, disaster planning considerations, and future research directions. There is some partial evidence to suggest that regardless of move status, increased neighborhood-level socioeconomic conditions may positively impact the mental health trajectories of disaster survivors. This finding is important in identifying and leveraging post-disaster windows of opportunities to provide post-disaster sheltering and housing assistance for disaster survivors. In line with previous studies, our findings demonstrate the importance of significant policy arenas for natural disasters, such as bolstering and investing in programs and policies related to affordable housing, housing restoration, and community revitalization [43,50]. Not only are these efforts critical in the immediate aftermath of a disaster, but our findings suggest the need for pre-disaster investment. Some findings from the Moving to Opportunity experiment, perhaps the largest in geographic scale random assignment, increased our understanding of the magnitude of neighborhood effects and arguably presented the strongest causal evidence to date [13,51]. Though many families ended up leaving their new neighborhoods over time, studies found that moving from high-poverty to lower-poverty neighborhoods led to long-term improvements in adult mental health and subjective well-being [51]. The near random displacement after Hurricane Katrina resulted in people relocating to and/or experiencing neighborhoods of different socioeconomic conditions which impacted people’s mental health over time.

We found that women who eventually returned to their pre-Katrina residence in the 10 years following the disaster were predicted to have relatively better mental health symptoms than those who moved away and did not return. This brings to light the question whether disaster survivors should return to their pre-disaster residence. Our findings show that individuals who returned had a greater number of economic resources to move back to their pre-disaster residence. This suggests increasing policy initiatives to create a more equal opportunity for all survivors to return to their neighborhoods (e.g., economic incentives), in addition to equitable rebuilding and restoration efforts for all disaster-affected communities. This also underscores the need to better understand the motivations for relocating back to one’s pre-disaster neighborhood [43]. There may be factors related to structural-level and individual-level social capital that are significant for disaster survivors, particularly women. Previous studies have established the linkage between social cohesion and mental health and disaster preparedness. Notably, a qualitative analysis of among a group of low-income mothers in New Orleans demonstrated the importance of their informal networks for coping with the evacuation and displacement after Katrina [52]. The disruption of women’s social networks, as a result of the displacement, led to challenges in rebuilding new social ties and recovering from economic losses in their new environments. In order to mitigate the disruption of informal networks when displacement is inevitable, increased funding and expansion of programs aimed to enhance community cohesion are critical both in the short- and long-term. However, it is equally critical to better understand the depth and utility of social capital among women. In light of our study’s findings, future research in the context of natural disasters should target the intersections of gender, social capital, geographic mobility, and mental health over time.

## 5. Conclusions

This study underscores the importance of understanding how neighborhood environments shape mental health over time among women survivors of Hurricane Katrina, a particularly vulnerable population who experienced post-disaster displacement and migration. We examined the impact of neighborhood-level social and economic conditions on individual-level mental health via a constructed measure of neighborhood SES. We found that increased neighborhood SES was associated with increased mental health. Furthermore, women who returned to their pre-disaster communities were predicted to have better mental health compared to women who did not return. Our findings justify further research to understand the mechanisms between displacement and mental health, in addition to exploring other dimensions of neighborhood social and economic conditions which are important for women’s mental health and well-being. In addition, our study also indicates the need to connect survivors to mental health services and disaster housing assistance programs both in the short- and long-term aftermath of a natural disaster.

## Figures and Tables

**Table 1:** Baseline characteristics by move status among GCAFH study population.

Characteristics	Overall *n* = 394	Stayed/Returned *n* = 69 [17.5%]	Moved *n* = 325 [82.5%]	*p*-Value
Age [mean, SD]	42.7 [13.8]	48.4 [13.5]	41.5 [13.6]	<0.001
Race				0.440
Black	200 [52.0%]	31 [45.6%]	169 [53.3%]	
White	165 [42.9%]	34 [50.0%]	131 [41.3%]	
Other	20 [5.2%]	3 [4.4%]	17 [5.4%]	
MCS [mean, SD]	39.6 [10.7]	38.7 [10.5]	39.8 [10.7]	0.470
PCS [mean, SD]	45.5 [14.5]	45.5 [15.8]	45.5 [14.2]	1.000
Marital Status				0.053
Non-Partnered	226 [62.4%]	32 [51.6%]	194 [64.7%]	
Partnered	136 [37.6%]	30 [48.4%]	106 [35.3%]	
Any children in household				0.457
No	144 [39.3%]	27 [43.6%]	117 [38.5%]	
Yes	222 [60.7%]	35 [56.5%]	187 [61.5%]	
Below poverty line status (Pre-Katrina)				<0.001
No	173 [46.0%]	46 [69.7%]	127 [41.0%]	
Yes	203 [54.0%]	20 [30.3%]	183 [59.0%]	
Below neighborhood MHI (Pre-Katrina)				0.041
No	112 [29.0%]	27 [39.1%]	85 [26.8 %]	
Yes	274 [71.0%]	42 [60.9%]	232 [73.2%]	
Neighborhood SES score (Pre-Katrina)	0.54 [0.14]	0.57 [0.13]	0.53 [0.14]	0.043

GCAFH: Gulf Coast Child and Family Health; SD: Standard Deviation; MCS: Mental Health Component Summary Score; PCS: Physical Health Component Summary Score; MHI: Median Household Income; SES: Socioeconomic Status.

**Table 2:** The results of unconditional means model (no predictors included) and unconditional growth model (time variable included) describing the changes in mental health scores across the GCAFH study sample.

	Unconditonal Means	Unconditional Growth
Fixed effect estimates		
MCS	44.5 [43.5, 45.5] *	42.0 [40.9, 43.2] *
Months		0.04 [0.03, 0.05] *
Variance components		
Census block groups	14.1 [7.1, 23.8]	6.4 [0.8, 15.0]
Between individual	50.7 [40.5, 62.6]	52.2 [41.9, 64.1]
Within individual	97.7 [89.9, 106.4]	97.7 [89.8, 106.4]
Intraclass correlation coefficients		
Neighborhood	0.087	0.041
Individuals within neighborhood	0.312	0.334
Model Fit		
Akaike Information Criterion	13,431.7	13,365.3

GCAFH: Gulf Coast Child and Family Health; MCS: Mental Health Component Summary Score. * *p* < 0.05.

**Table 3:** Nested models describing the associations between neighborhood socioeconomic status and MCS score after Hurricane Katrina among GCAFH study sample.

	Intermediate Model	Final Model
Fixed Effect Estimates		
Intercept	53.0 [46.4, 59.5] *	45.5 [35.7, 55.0] *
Neighborhood SES score	6.5 [0.05, 13.0]	19.6 [5.8, 33.7] *
Move status		
Stayed or returned	Reference	Reference
Moved	−1.7 [−4.1, 0.8]	7.3 [−1.4, 16.1]
Neighborhood SES score * Move status	-	−15.4 [−30.2, −1.1] *
Age	−0.05 [−0.1, 0.02]	−0.05 [−0.1, 0.02]
Race		
Black	Reference	Reference
White	−2.0 [−4.2, 0.1]	−2.0 [−4.1, 0.2]
Other	−1.2 [−5.1, 2.8]	−1.2 [−5.1, 2.8]
Children in household		
No	Reference	Reference
Yes	−2.0 [−4.1, 0.05]	−2.0 [−0.2, −0.1]
PCS	−0.2 [−0.2, −0.1] *	−0.2 [−0.2, −0.1] *
Neighborhood SES score (pre-Katrina)	2.2 [−5.7, 10.0]	2.0 [−5.8, 9.8]
Poverty line at baseline (pre-Katrina)	0.4 [−1.9, 2.7]	0.4 [−1.8, 2.7]
Poverty line	−1.5 [−3.1, 0.08]	−1.4 [ −3.0, 0.2]
Below neighborhood MHI (pre-Katrina)	−2.0 [−4.5, 0.5]	−2.0 [−4.5, 0.4]
Below neighborhood MHI	−1.7 [−3.4, −0.1] *	−1.8 [−3.5, −0.2] *
Months	0.03 [0.01, 0.04] *	0.03 [0.01, 0.04] *
Variance components		
Census block groups	9.6 [1.5, 20.2]	8.9 [1.2 19.0]
Between individual	54.3 [41.6, 65.9]	53.0 [40.4, 64.4]
Within individual	90.7 [82.1, 99.9]	91.3 [82.6, 100.5]
Intraclass correlation coefficients		
Neighborhood	0.062	0.058
Individuals within neighborhood	0.351	0.346
Model Fit		
Akaike Information Criterion	11,237.4	11,229.3

MCS: Mental Health Component Summary; GCAFH: Gulf Coast Child and Family Health; SES: Socioeconomic Status; PCS: Physical Health Component Summary; MHI: Median Household Income; * *p* < 0.05.

## Data Availability

The data presented in this study are available on request from the corresponding author. The data are not publicly available due to the privacy and confidentiality of research participants.
